# Cancer treatment and survival among cervical cancer patients living with or without HIV in South Africa

**DOI:** 10.1016/j.gore.2022.101069

**Published:** 2022-09-21

**Authors:** Yannick Q. Turdo, Yann Ruffieux, Tebatso M.G. Boshomane, Hannes Mouton, Katayoun Taghavi, Andreas D. Haas, Matthias Egger, Gary Maartens, Eliane Rohner

**Affiliations:** a Institute of Social and Preventive Medicine, University of Bern, Bern, Switzerland; b Department of Nuclear Medicine, University of Pretoria & Steve Biko Academic Hospital, Pretoria, South Africa; c Division of Clinical Pharmacology, Department of Medicine, University of Cape Town, Cape Town, South Africa; d Population Health Sciences, Bristol Medical School, University of Bristol, Bristol, UK; e Centre for Infectious Disease Epidemiology and Research, University of Cape Town, Cape Town, South Africa

**Keywords:** Cervical cancer, HIV/AIDS, Survival, South Africa

## Abstract

**Objective:**

To compare cancer treatment and all-cause mortality between HIV-positive and HIV-negative cervical cancer patients in South Africa.

**Methods:**

We assessed cancer treatment and all-cause mortality in HIV-positive and HIV-negative cervical cancer patients who received cancer treatment within 180 days of diagnosis using reimbursement claims data from a private medical insurance scheme in South Africa between 01/2011 and 07/2020. We assessed treatment provision using logistic regression and factors associated with all-cause mortality using Cox regression. We assigned missing values for histology and ethnicity using multiple imputation.

**Results:**

Of 483 included women, 136 (28 %) were HIV-positive at cancer diagnosis (median age: 45.7 years), and 347 (72 %) were HIV-negative (median age: 54.1 years). Among 285 patients with available ICD-O-3 morphology claims codes, the proportion with cervical adenocarcinoma was substantially lower in HIV-positive (4 %) than in HIV-negative patients (26 %). Most HIV-positive patients (67 %) were on antiretroviral therapy at cancer diagnosis. HIV-positive patients were more likely to receive radiotherapy (adjusted odds ratio [aOR] 1.90, 95 % confidence interval [CI] 1.05–3.45) or chemotherapy (aOR 2.02, 95 %CI 0.92–4.43) and less likely to undergo surgery (aOR 0.53, 95 %CI 0.31–0.90) than HIV-negative patients. HIV-positive patients were at a higher risk of death from all causes than HIV-negative patients (adjusted hazard ratio 1.52, 95 %CI 1.06–2.19). Other factors associated with higher all-cause mortality included age > 60 years and metastases at diagnosis.

**Conclusions:**

HIV-positive cervical cancer patients in South Africa had higher all-cause mortality than HIV-negative patients which could be explained by differences in tumour progression, clinical care, and HIV-specific mortality.

## Highlights

• Analysis of cervical cancer treatment and survival by HIV status using reimbursement claims data from South Africa.
• HIV-positive patients were more likely to receive radio- and chemotherapy and less likely to undergo surgery.
• HIV-positive cervical cancer patients were at higher risk of death from all causes than HIV-negative patients.

## 1 Introduction

Cervical cancer causes approximately 342,000 annual deaths, making it the fourth most common cause of cancer-related mortality among women worldwide. (Ferlay et al., 2018) Women in sub-Saharan Africa bear a disproportionate share of the global cervical cancer burden. (Ferlay et al., 2018) In South Africa, cervical cancer is the second most common cancer with approximately 11,000 new cases every year and the most frequent cause of cancer-related death among women. (Ferlay et al., 2018) Infection with high-risk human papillomavirus (HPV) subtypes is the most important etiological factor in the development of cervical cancer. HPV is detectable in virtually all cervical cancer biopsies. (Bosch et al., 1995, Walboomers et al., 1999) While HPV vaccination, screening, and treatment of pre-cancerous lesions led to a decline in cervical cancer incidence in high-income countries, low- and middle-income countries with limited access to preventive measures still bear a high burden of cervical cancer. (Denny et al., 2017) Women living with HIV (WLWH) are approximately six-times more likely to develop cervical cancer than women without HIV. (Stelzle et al., 2021) HIV infection is associated with an increased risk of HPV acquisition and persistence. (Massad et al., 2014, Liu et al., 2018) HIV-induced immunosuppression also increases the oncogenic effects of HPV. (IARC Working Group on the Evaluation of Carcinogenic Risks to Humans, 2012) In 1993, invasive cervical cancer was classified as one of three AIDS-defining cancers alongside Kaposi sarcoma and non-Hodgkin lymphoma. (Ward, 1992, Castle et al., 2021) South Africa has one of the highest HIV prevalence worldwide. In 2020, one in four women aged between 15 and 49 years were living with HIV, making this population especially vulnerable to cervical cancer. (AIDSinfo | UNAIDS).

Studies in low- and middle-income countries have reported discrepant findings on the association of HIV status with cervical cancer stage at diagnosis, treatment, and survival. Some studies found a higher risk of late cancer diagnosis among HIV-positive cervical cancer patients. (Simonds et al., 2012, Simonds et al., 2018) In contrast, others reported no differences in cancer stage by HIV status. (Dryden-Peterson et al., 2016, Ferreira et al., 2017, Wu et al., 2020) Furthermore, HIV-positive cervical cancer patients might be less likely to receive chemotherapy because of low CD4 cell counts and more likely to experience treatment interruptions. (Simonds et al., 2012, du Toit and Kidd, 2015, Simonds et al., 2015) In contrast, in patients with well-controlled HIV disease, cervical cancer treatment modalities and completion rates do not vary by HIV status. (Dryden-Peterson et al., 2016, Grover et al., 2018, MacDuffie et al., 2021) Several studies found a higher risk of cancer relapse and lower survival among HIV-positive than HIV-negative cervical cancer patients. (Simonds et al., 2018, Dryden-Peterson et al., 2016, Ferreira et al., 2017, Wu et al., 2020, Mangena et al., 2015) Others did not find differences in cervical cancer survival based on HIV status. (Grover et al., 2018, MacDuffie et al., 2021, Thokanit et al., 2020, Jemu et al., 2018, Gizaw et al., 2017).

We assessed differences in cancer treatment and all-cause survival in cervical cancer patients living with and without HIV in South Africa using reimbursement claims data from a large private medical aid scheme operating in South Africa.

## 2 Methods

### 2.1 Data source

We used patient-level data from a medical aid scheme database including inpatient and outpatient reimbursement claims data between 1 January 2011 and 1 July 2020. The claims data were coded using International Statistical Classification of Diseases and Related Health Problems (ICD-10), International Classification of Diseases for Oncology (ICD-O-3), Current Procedural Terminology (CPT), Anatomical Therapeutic Chemical (ATC), and National Reference Price List (NRPL) codes. The database also included laboratory test results between 2016 and 2020 and demographic information. Information on vital status and date of death was obtained from the database and completed through a linkage with the South African National Population Register (NPR). The Human Research Ethics Committee of the University of Cape Town, South Africa, and the Cantonal Ethics Committee of the Canton of Bern, Switzerland, provided ethical approval for this analysis.

### 2.2 Inclusion criteria and definitions

We included women who had been diagnosed with invasive cervical cancer between 1 January 2011 and 1 July 2020 and who received cancer treatment within 180 days of a cervical cancer diagnosis. We considered women to be cervical cancer patients if they had at least two C53 ICD-10 codes recorded on separate days after enrolment into the cohort. We defined the date of a cervical cancer diagnosis as the date of the first C53 code in the database. HIV status was determined based on the following indicators: ICD-10 codes (B20-B24), laboratory data (positive HIV test or availability of HIV viral load, CD4 cell count, or CD4 percentage measurements), ATC codes for antiretroviral therapy (ART), and identifiers linking patients to the Aid for AIDS (AfA) HIV disease management program. Patients were regarded as HIV-positive if they had two or more HIV indicators, with the first indicator recorded before or up to six months after the cervical cancer diagnosis. Women without any HIV indicators were considered HIV-negative. We excluded women with only one HIV indicator, women with a first HIV indicator recorded more than six months after the cervical cancer diagnosis, women with missing information on date of birth, women without any follow-up time after cervical cancer diagnosis, and women who were not included in the linkage with the NPR.

We determined ART status, cancer stage (localised versus metastasised), histological tumour type, and cancer treatment (radiotherapy, chemotherapy, and surgery) based on ICD-10, ICD-O-3, CPT, ATC, and NRPL codes. Patients were considered on ART at cervical cancer diagnosis if ATC codes for ART appeared before cancer diagnosis. We defined metastasised cancer stage at cancer diagnosis as the appearance of an ICD-10 code for secondary malignancy (C77-C79.9) or an ICD-O-3 code for metastatic behaviour (/6) within 30 days of diagnosis. All other cancer cases were defined as localised. We assessed ICD-O-3 morphology claims codes from 30 days before until 90 days after cervical cancer diagnosis to evaluate tumour histology. CPT and NRPL codes for cervical cancer surgery (i.e., hysterectomy and trachelectomy) recorded within 90 days before cervical cancer diagnosis were carried forward to the day of diagnosis to capture procedures that preceded the diagnosis. Supplementary Table S1 provides a detailed list of codes used for data extraction. We assessed CD4 cell counts, HIV RNA viral loads, and nadir haemoglobin (Hb) levels within a time window of 90 days before to 90 days after cancer diagnosis for patients diagnosed with cervical cancer between 2016 and 2020. We imputed missing values on ethnicity and histological tumour type using multiple imputation by chained equations. We assumed values were missing at random and pooled analyses from 50 sets of imputations using Rubin’s rule. (van Buuren and Groothuis-Oudshoorn, 2011) The imputation model included HIV status, age, cancer stage, calendar year at cancer diagnosis, cancer treatment (radiotherapy, chemotherapy, and surgery), and death.

### 2.3 Statistical analysis

We compared patient demographics and clinical characteristics at cervical cancer diagnosis between HIV-negative and HIV-positive patients, using Student’s *t*-test for continuous and Pearson’s chi-squared or Fisher’s exact tests for categorical variables, where applicable. We used separate logistic regression models to estimate odds ratios (ORs) of receiving a specific type of cervical cancer treatment (i.e., radiotherapy, chemotherapy, or surgery) based on different patient characteristics. The multivariable logistic regression model included HIV status, age group, ethnicity, tumour histology, and cancer stage. We compared time from cancer diagnosis to cancer treatment and all-cause survival by HIV status using Kaplan-Meier curves. We also produced age-standardized survival curves. We used Cox proportional hazards models to analyse factors associated with all-cause mortality. P-values below 0.05 were used as a cut-off for testing the proportional hazards assumption. The multivariable Cox model included HIV status, age group, ethnicity, tumour histology, and cancer stage. We generated Kaplan-Meier and age-standardized survival curves stratified by cancer stage or ART. In all time-to-event analyses, we right-censored follow-up at transfer from the medical aim scheme, death, or database closure (1 July 2020). In sensitivity analyses, we ran the logistic and the Cox regression models using categorical variables for missing data instead of imputed data for ethnicity and tumour histology. Statistical analysis was performed using R 4.1.2 (R Foundation for Statistical Computing, Vienna, Austria) and STATA version 16.1 (StataCorp, College Station, Texas, USA).

## 3 Results

**Fig. 1:**
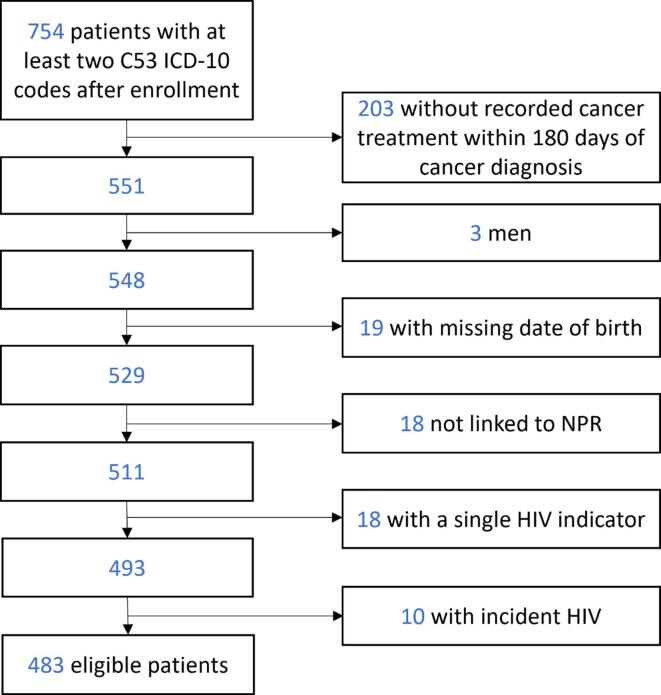
Identification of study population. The flow diagram shows the number of included and excluded patients. ICD-10, International Statistical Classification of Diseases and Related Health Problems; NPR, South African National Population Register.

Between January 2011 and July 2020, 754 women had a cervical cancer diagnosis recorded in the medical claims database. We excluded 203 patients (148 HIV-negative, 49 HIV-positive, and six with a single HIV indicator) because they had no cancer treatment recorded within six months after the cervical cancer diagnosis. Another 68 patients were excluded for reasons listed in Fig. 1.

Among the 483 cervical cancer patients included in the analysis, 136 patients (28 %) were HIV-positive, and 347 (72 %) HIV-negative (Table 1). HIV-positive patients were younger at cervical cancer diagnosis (median age: 45.7 years, interquartile range [IQR] 39.8–49.5) than HIV-negative patients (54.1 years, IQR 44.7–63.7). Among 404 patients with known ethnicity, 96 % of HIV-positive cervical cancer patients were Black compared with 61 % among HIV-negative cervical cancer patients. Among 285 patients with available information on tumour histology, squamous cell carcinoma (SCC) was the predominant subtype in both HIV-positive (93 %) and HIV-negative patients (68 %). The percentage of adenocarcinoma was much lower in HIV-positive (4 %) than in HIV-negative patients (26 %). At cervical cancer diagnosis 67 % of HIV-positive patients were on ART. Median nadir Hb was 9.3 g/dL (IQR 7.7–10.4) in HIV-positive patients and 10.1 g/dL (IQR 8.5–15.1) in HIV-negative cervical cancer patients diagnosed between 2016 and 2020. However, information on nadir Hb at cervical cancer diagnosis was missing for 81 % of HIV-negative and 25 % of HIV-positive patients. CD4 cell count and HIV RNA viral load measurements were missing for 57 % and 53 % of HIV-positive patients, respectively. Among those with data, 76 % had an HIV RNA viral load < 1000 copies/mL at cancer diagnosis. At cancer diagnosis, the median CD4 cell count was 408 cells/ µL (IQR 327–571).

**Table 1:** Patient demographic and clinical characteristics at cervical cancer diagnosis, stratified by HIV status.

**Characteristics**	**HIV-negative**	**HIV-positive**	**Total**	**p-value**
**Number of patients**	347	136	483	
**Median age (IQR) [years]**	54.1 [44.7, 63.7]	45.7 [39.8, 49.5]	50.6 [42.2, 59.6]	<0.001
**Age category**				<0.001
<40 years	53 (15.3)	38 (27.9)	91 (18.8)	
40–59 years	180 (51.9)	94 (69.1)	274 (56.7)	
≥60 years	114 (32.9)	4 (2.9)	118 (24.4)	
**Ethnicity**				<0.001
Black	178 (60.8)	107 (96.4)	285 (70.5)	
White	79 (27.0)	1 (0.9)	80 (19.8)	
Indian/Asian	17 (5.8)	1 (0.9)	18 (4.5)	
Mixed ancestry	19 (6.5)	2 (1.8)	21 (5.2)	
Missing	54	25	79	
**Calendar year at cancer diagnosis category**				0.088
2011–2015	172 (49.6)	55 (40.4)	227 (47.0)	
2016–2020	175 (50.4)	81 (59.6)	256 (53.0)	
**Cancer stage at diagnosis**				0.484
Localised	307 (88.5)	124 (91.2)	431 (89.2)	
Metastasised	40 (11.5)	12 (8.8)	52 (10.8)	
**Histological tumour type**				<0.001
Squamous cell carcinoma	129 (67.9)	88 (92.6)	217 (76.1)	
Adenocarcinoma	50 (26.3)	4 (4.2)	54 (18.9)	
Other	11 (5.8)	3 (3.2)	14 (4.9)	
Missing	157	41	198	
Median nadir Hb level (IQR) [g/dL]*	10.1 [8.5, 12.1]	9.3 [7.7, 10.4]	9.5 [8.0, 10.7]	0.040
Missing	141 (80.6)	20 (24.7)	161 (62.9)	<0.001
**On ART at cancer diagnosis**	–	91 (66.9)	–	
HIV RNA viral load*				
< 1000 copies/mL	–	29 (76.3)	–	
≥ 1000 copies/mL	–	9 (23.7)	–	
Missing		43		
Median CD4 cell count (IQR) [cells/µL]*	–	408 [327, 571]	–	
Missing		46 (56.8)		

* For patients diagnosed with cervical cancer in 2016 or later (175 HIV-negative, 81 HIV-positive).

ART, antiretroviral therapy; Hb, haemoglobin; IQR, interquartile range.

Results are presented as numbers and percentages if not otherwise stated.

### 3.1 Cancer treatment

**Fig. 2:**
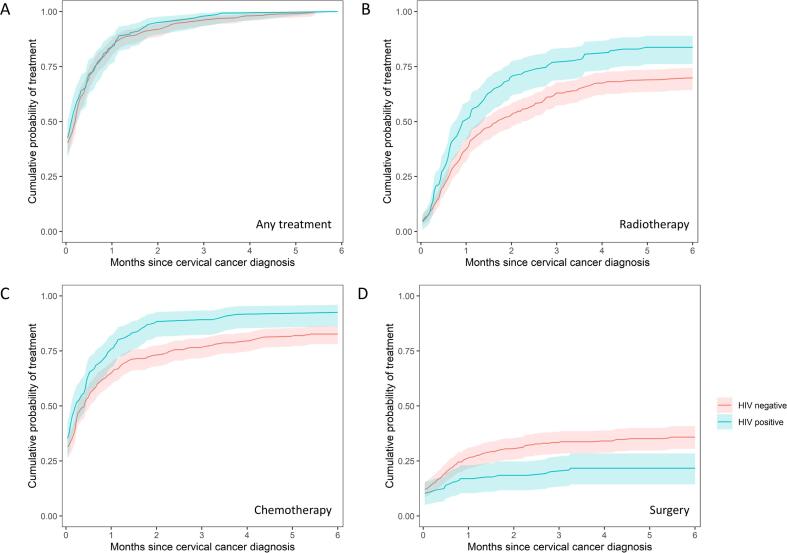
Cumulative probability of receiving any treatment (Panel A), radiotherapy (Panel B), chemotherapy (Panel C), or surgery (Panel D) within 6 months of cervical cancer diagnosis, stratified by HIV status.

Overall, 150 (31 %) cervical cancer patients had surgical treatment, 349 (72 %) patients underwent radiotherapy, and 409 (85 %) patients received chemotherapy. After cervical cancer diagnosis, 404 patients (84 %) received any cancer treatment within one month and 447 patients (93 %) within two months. Time to any treatment was similar in HIV-positive and HIV-negative patients (Fig. 2 Panel A). However, HIV-positive patients were more likely to receive radiotherapy and chemotherapy and less likely to receive surgery than HIV-negative patients (Fig. 2 Panels B-D).

For 147 patients (30 %), the first medical claims code for cervical cancer coincided with the initiation of chemotherapy. Table 2 shows the adjusted odds ratios (aOR) for factors associated with receiving a specific cancer treatment.

**Table 2:** Adjusted odds ratios for receiving cancer treatment within 6 months after cervical cancer diagnosis.

	**Patients treated (N)**	**aOR for radiotherapy** **(95 % CI)**	**Patients treated (N)**	**aOR for chemotherapy** **(95 % CI)**	**Patients treated (N)**	**aOR for surgery** **(95 % CI)**
**Characteristic**						
**HIV status**						
Negative	236	1	284	1	121	1
Positive	113	1.90 (1.05–3.45)	125	2.02 (0.92–4.43)	29	0.53 (0.31–0.90)
**Age category**						
<40 years	63	1	73	1	38	1
40–59 years	203	1.39 (0.78–2.49)	238	1.89 (0.96–3.76)	81	0.54 (0.32–0.92)
≥60 years	83	2.18 (1.07–4.45)	98	1.89 (0.85–4.24)	31	0.33 (0.17–0.65)
Ethnicity*						
Black	–	1	–	1	–	1
Other**	–	1.16 (0.66–2.05)	–	0.80 (0.43–1.51)	–	1.48 (0.89–2.44)
Histological tumour type*						
Squamous cell carcinoma	–	1	–	1	–	1
Adenocarcinoma	–	0.24 (0.12–0.46)	–	0.45 (0.20–0.99)	–	1.62 (0.84–3.11)
Other	–	0.19 (0.07–0.49)	–	0.15 (0.05–0.43)	–	2.77 (1.05–7.31)
**Cancer stage at diagnosis**						
Localised	318	1	361	1	141	1
Metastasised	31	0.62 (0.32–1.20)	48	3.07 (1.02–9.27)	9	0.39 (0.18–0.85)

* Missing values for ethnicity and histological tumour type were imputed using multiple imputation.

** Other include White, Indian/Asian, and Mixed ancestry ethnicities.

aOR, adjusted odds ratio; CI, confidence interval.

HIV-positive patients were more likely to receive radiotherapy (aOR 1.90, 95 % confidence interval [CI] 1.05–3.45) or chemotherapy (aOR 2.02, 95 %CI 0.92–4.43) and less likely to receive surgery (aOR 0.53, 95 %CI 0.31–0.90) than HIV-negative patients. Similarly, patients aged 60 years or older were more likely to receive radiotherapy (aOR 2.18, 95 %CI 1.07–4.45) or chemotherapy (aOR 1.89, 95 %CI 0.85–4.24) and less likely to receive surgery (aOR 0.33, 95 %CI 0.17–0.65) than patients under the age of 40 years. Patients with cervical adenocarcinoma were less likely to receive radiotherapy (aOR 0.24, 95 %CI 0.12–0.46) and chemotherapy (aOR 0.45, 95 %CI 0.20–0.99) but more likely to undergo surgical treatment (aOR 1.62, 95 %CI 0.84–3.11) than patients with SCC. Ethnicity did not correlate with cancer treatment modality (Table 2). Finally, patients with metastasised cervical cancer at diagnosis were much more likely to receive chemotherapy (aOR 3.07, 95 %CI 1.02–9.27) and less likely to undergo radiotherapy (aOR 0.62, 95 %CI 0.32–1.20) or surgery (aOR 0.39, 95 %CI 0.18–0.85) than patients with localised cancer stage. Unadjusted odds ratios (OR) were broadly similar, except that chemotherapy and radiotherapy did not show a clear association with age in univariable analyses (Supplementary Table S2).

### 3.2 Survival

**Fig. 3:**
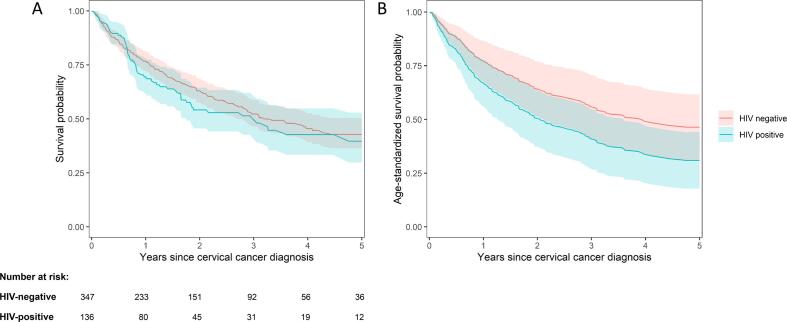
Kaplan-Meier (Panel A) and age-standardized overall survival curves (Panel B), stratified by HIV status.

During 1046 person-years of follow-up, 63 (46 %) HIV-positive and 149 (43 %) HIV-negative cervical cancer patients died. The median survival after cervical cancer diagnosis was 1092 days (3.0 years) among HIV-positive and 1166 days (3.2 years) among HIV-negative patients. Fig. 3A shows a Kaplan-Meier analysis for overall survival stratified by HIV status over five years, with survival curves starting to diverge around 9–12 months after cancer diagnosis. Two-year survival was 54 % (95 %CI 46–64 %) among HIV-positive and 63 % (95 %CI 58–69 %) among HIV-negative patients. Five-year survival was 40 % (95 %CI 30–53 %) among HIV-positive and 43 % (95 %CI 36–50 %) among HIV-negative patients. Lower overall survival in HIV-positive than HIV-negative patients was more apparent in the age-standardized survival curves (Fig. 3B).

In univariable analyses, the risk of death was 18 % higher among HIV-positive than HIV-negative cervical cancer patients (hazard ratio [HR] 1.18, 95 %CI 0.88–1.58). After adjusting for age, ethnicity, tumour histology, and cancer stage at diagnosis, the association of HIV status with all-cause mortality became stronger (adjusted hazard ratio [aHR] 1.52, 95 %CI 1.06–2.19). Older age (≥60 versus < 40 years) was also associated with higher all-cause mortality in univariable (HR 2.01, 95 %CI 1.35–3.02) and multivariable analyses (aHR 2.24, 95 %CI 1.43–3.50). Ethnicity did not show a clear association with all-cause mortality (Table 3). In univariable analyses, patients with cervical adenocarcinoma had higher risk of dying than patients with SCC (HR 1.45, 95 %CI 1.03–2.06). The association between histological subtype and all-cause mortality was weaker in the multivariable analysis (aHR 1.28, 95 %CI 0.86–1.91). The proportional hazards assumption did not hold (p-value from Schoenfeld residual test = 0.005) for cancer stage at diagnosis with a steep drop in survival probabilities for those with metastasised cancer early after diagnosis (Supplementary Figure S1). We found a fourfold higher mortality among patients with metastasised cancer at diagnosis than those with localised cancer at diagnosis during the first nine months of follow-up after cervical cancer diagnosis (aHR 3.94, 95 %CI 2.47–6.28), whereas the association of cancer stage with all-cause mortality became less pronounced more than nine months after cancer diagnosis (aHR 1.37, 95 %CI 0.73–2.58). All-cause survival was slightly better in HIV-positive patients on ART at cancer diagnosis than in those who had not initiated ART, but uncertainty around the estimates was large (Supplementary Figure S2).

**Table 3:** Unadjusted and adjusted hazard ratios for all-cause mortality after cervical cancer diagnosis.

		**Unadjusted**	**Adjusted**
**Characteristic**	**Deaths (N)**	**Hazard Ratio (95 % CI)**	**Hazard Ratio (95 % CI)**
**HIV status**			
Negative	149	1	1
Positive	63	1.18 (0.88–1.58)	1.52 (1.06–2.19)
**Age category**			
<40 years	36	1	1
40–59 years	105	1.07 (0.73–1.56)	1.11 (0.75–1.64)
≥60 years	71	2.01 (1.35–3.02)	2.24 (1.43–3.50)
Ethnicity*			
Black	–	1	1
Other**	–	0.73 (0.52–1.03)	0.72 (0.49–1.06)
Histological tumour type*			
Squamous cell carcinoma	–	1	1
Adenocarcinoma	–	1.45 (1.03–2.06)	1.28 (0.86–1.91)
Other	–	1.53 (0.81–2.91)	1.60 (0.84–3.07)
**Cancer stage (mortality up to 9 months)**			
Localised	67	1	1
Metastasised	27	4.37 (2.79–6.84)	3.94 (2.47–6.28)
**Cancer stage (mortality after 9 months)**			
Localised	107	1	1
Metastasised	11	1.54 (0.83–2.87)	1.37 (0.73–2.58)

* Missing values for ethnicity and histological tumour type were imputed using multiple imputation.

** Other include White, Indian/Asian, and Mixed ancestry ethnicities.

CI, confidence interval.

The adjusted hazard ratios for HIV status, age, and ethnicity, are obtained from a single model stratified with respect to the cancer stage variable. The hazard ratios (both adjusted and unadjusted) for cancer stage are obtained from separate models, one censoring time-at-risk at 9 months, and another starting time-at-risk at 9 months.

### 3.3 Sensitivity analyses

Sensitivity analyses that included patients with missing values on ethnicity and histological tumour type as a separate category yielded similar results for cancer treatment (Supplementary Table S3) and all-cause mortality (Supplementary Table S4).

## 4 Discussion

In South Africa, HIV-positive cervical cancer patients receiving cancer treatment within six months of diagnosis, had a 50 % higher all-cause mortality rate than their HIV-negative counterparts after adjusting for age, ethnicity, histological tumour type, and metastases at diagnosis. Crude all-cause five-year survival was 40 % for HIV-positive and 43 % for HIV-negative patients. HIV-positive patients were more likely to be treated with radiotherapy and chemotherapy and less likely to receive surgery than HIV-negative patients. Cervical adenocarcinomas were substantially less common in HIV-positive (4 %) than in HIV-negative patients (26 %).

The strengths of our study include the relatively large sample size and proportion of HIV-positive patients compared to most other studies exploring cervical cancer survival in African populations. (Simonds et al., 2018, Dryden-Peterson et al., 2016, Wu et al., 2020, Grover et al., 2018, Mangena et al., 2015, Jemu et al., 2018) Furthermore, we verified the vital status of the included cervical cancer patients through a linkage with the NPR. However, our findings must be considered in light of several limitations. Medical claims data may be unreliable and confirmatory conditions are required to identify credible incident cancer cases. (Czwikla et al., 2017) We addressed this issue by restricting the dataset to women who had at least two ICD-10 codes for cervical cancer and required additional codes for cancer treatment to increase the specificity of our algorithm. For HIV status, we required patients to have two HIV indicators and excluded patients with ambiguous information. We assumed that patients without any HIV indicators were HIV-negative, but some of these patients may have had undiagnosed HIV. Furthermore, HIV-related laboratory results were not available for patients diagnosed with cervical cancer before 2016. However, given that we defined HIV status based on several indicators, we expect the impact of the missing laboratory data on HIV misclassification to be small. Our results on Hb, CD4 cell count, and HIV RNA viral load around cervical cancer diagnosis obtained for patients diagnosed between 2016 and 2020 may not necessarily be representative of cervical cancer patients diagnosed before 2016. Additionally, laboratory results were missing for a substantial proportion of patients diagnosed with cervical cancer between 2016 and 2020. For ethnicity and histological tumour type, we used multiple imputation to assign values where information was missing. We did not have data on International Federation of Gynaecology and Obstetrics (FIGO) cancer stage and could only distinguish between localised and metastasised cervical cancer based on ICD-10 and ICD-O-3 codes. While the medical claims data allowed us to examine the type of cancer treatment received, we were unable to consider in-depth treatment information such as timing, completion, and dosage. We did not have detailed cause of death information and were therefore limited to analysing all-cause rather than cancer-specific mortality. Finally, our study covers a privately insured population that is not necessarily representative of the general South African population.

Cervical cancer management is based on FIGO cancer stage and treatment options include surgery, chemotherapy, and radiotherapy (external beam radiotherapy or brachytherapy). (Cohen et al., 2019) In general, early stage cervical cancer (FIGO I-IIA) is treated surgically with adjuvant and neoadjuvant radio- and chemotherapy options. In contrast, advanced stage tumours (FIGO IIB-IVA) are treated non-surgically using chemo-radiation therapy. (Cohen et al., 2019) A study of 383 patients diagnosed with stage IB1-IIIB cervical cancer between 2007 and 2011, who received radiotherapy in South Africa, found that HIV-positive patients presented with more advanced stage cancer and were less likely to complete radiotherapy and chemotherapy. (Simonds et al., 2012) In an overlapping cohort of 213 cervical cancer patients treated between 2009 and 2011 HIV-positive patients were more likely to experience haematological toxicity and less likely to complete chemotherapy. (Simonds et al., 2015) A smaller study of cervical cancer patients receiving cisplatin-based chemo-radiation therapy between 2012 and 2016 in Portugal reported an increased risk of neutropenia among HIV-positive patients and lower rates of treatment completion compared with HIV-negative patients. (Vendrell et al., 2018) In our study we were unable to evaluate completion of cancer treatment, but found that time to treatment initiation was virtually identical in HIV-positive and HIV-negative patients. We however observed differences in treatment type where HIV-positive patients were more likely to receive radiotherapy or chemotherapy and less likely to receive surgery. We hypothesise that this might be due to HIV-positive patients presenting with more advanced stages of localised cervical cancer when surgery is no longer recommended, (Simonds et al., 2012, Simonds et al., 2018) although some studies have found no differences in cancer stage by HIV status. (Dryden-Peterson et al., 2016, Ferreira et al., 2017, Wu et al., 2020).

Our findings support cohort studies in low- and middle-income countries that found higher mortality rates among HIV-positive than HIV-negative cervical cancer patients. (Simonds et al., 2012, Simonds et al., 2018, Wu et al., 2020) A South African study among cervical cancer patients who underwent radiotherapy with curative intent reported higher rates of all-cause mortality among HIV-positive than HIV-negative patients, even when excluding patients with low CD4 cell counts (<200 cells/µL). (Simonds et al., 2018) Reported two-year survival among HIV-negative patients (62 %) was similar to the estimate in our study (63 %), whereas HIV-positive patients had lower two-year survival (42 %) than those in our study (54 %). (Simonds et al., 2018) A study among 348 cervical cancer patients in Botswana found that a positive HIV status nearly doubled the adjusted risk of death. (Dryden-Peterson et al., 2016) A Ugandan study of 149 cervical cancer patients showed a 60 % increased risk of death in HIV-positive compared with HIV-negative patients in univariable analysis. However, the association between positive HIV status and all-cause mortality was attenuated after adjustment for age and cancer stage. (Wu et al., 2020) Additionally, the study found that crude all-cause one-year survival was similar in HIV-positive (65 %) and HIV-negative (69 %) patients but started diverging thereafter with HIV-positive patients having worse survival. We and others also observed a divergence in all-cause survival between HIV-positive and HIV-negative patients at 6–12 months after cancer diagnosis. (Dryden-Peterson et al., 2016, Wu et al., 2020, Mangena et al., 2015) Only few studies in high-income countries compared cervical cancer survival by HIV status but showed similar trends. A large US study found higher rates in cancer specific mortality among HIV-positive cervical cancer patients than HIV-negative patients, after adjusting for age at cancer diagnosis, ethnicity, year of cancer diagnosis and receipt of cancer treatment. (Coghill et al., 2015) A smaller study based in France found that five-year survival was lower among HIV-positive (66 %) than HIV-negative cervical cancer patients (74 %). However, five-year survival for both groups was much higher than in our study. (Grabar et al., 2019).

Other studies have not found differences in all-cause survival by HIV status. (Grover et al., 2018, MacDuffie et al., 2021, Thokanit et al., 2020, Jemu et al., 2018, Gizaw et al., 2017) A study of cervical cancer patients initiating curative chemo-radiation therapy in Botswana reported similar two- and five-year survival in HIV-positive and HIV-negative patients. (Grover et al., 2018, MacDuffie et al., 2021) The authors attributed this finding to the well-controlled HIV disease in their study population with a relatively high median CD4 cell count at cervical cancer diagnosis (481 cells/µL) and virtually all HIV-positive patients (96 %) receiving ART. In most other studies, about 60–80 % of HIV-positive patients were on ART at cervical cancer diagnosis, and CD4 cell counts were generally lower (<400 cells/µL). (Simonds et al., 2018, Dryden-Peterson et al., 2016, Ferreira et al., 2017, Wu et al., 2020) In our study, 67 % of HIV-positive patients had initiated ART before cervical cancer diagnosis, and the median CD4 cell count at cancer diagnosis was 408 cells/µL. However, the median CD4 cell count should be interpreted cautiously due to the large proportion of missing laboratory results in our study. Two studies in South Africa and Thailand, which did not find an association between HIV status and all-cause survival, included low numbers of HIV-positive cervical cancer patients, and potentially lacked the statistical power to detect a difference. (Thokanit et al., 2020, Jemu et al., 2018) Finally, a study based on routinely collected data in Ethiopia found no clear association between HIV status and survival among 1655 cervical cancer patients, 139 of whom were confirmed HIV-positive and 368 were confirmed HIV-negative. (Gizaw et al., 2017).

Besides HIV status, we found that older age and metastases at diagnosis were both strongly associated with higher all-cause mortality among cervical cancer patients. In contrast, there was no clear association between all-cause mortality and ethnicity or histological tumour type. Interestingly, in our study, the distribution of histological tumour subtypes differed between HIV-positive and HIV-negative cervical cancer patients. Although SCC was the predominant subtype in both patient groups, the percentage of cervical adenocarcinoma was much lower in HIV-positive (4 %) than in HIV-negative patients (26 %). Other studies have also found lower percentages of adenocarcinoma in HIV-positive than HIV-negative cervical cancer patients. (Simonds et al., 2012, Simonds et al., 2018, Dryden-Peterson et al., 2016, Ferreira et al., 2017, Grover et al., 2018, Rositch et al., 2022) A US-based study comparing cervical cancer incidence rates in WLWH to the general population reported a larger standardised incidence ratio for SCC (3.62, 95 % CI 3.31–3.94) than for adenocarcinoma (1.47, 95 % CI 1.03–2.05). (Rositch et al., 2022) It is not well understood why the distribution of histological subtypes varies by HIV status. Differences in HPV prevalence may play a role as HPV can be detected in virtually all cervical SCC but only 71 % of cervical adenocarcinomas. (Andersson et al., 2001) HIV increases the risk of persistent HPV infection, and thus, WLWH may be more prone to developing SCC than adenocarcinoma. (Stelzle et al., 2021, Massad et al., 2014, Liu et al., 2018) Surprisingly, HPV18, the most common HPV genotype in cervical adenocarcinoma may be more prevalent in WLWH than in women living without HIV. (Li et al., 2011, Clifford et al., 2006).

The association between positive HIV status and all-cause mortality in our study is not explained by differential access to cancer treatment as we only included patients who received cancer treatment within six months of diagnosis. Furthermore, the time to any cancer treatment was similar in HIV-positive and HIV-negative patients. However, it is possible that HIV-positive patients were diagnosed in more advanced localised cervical cancer stages than HIV-negative patients due to delays in screening or more rapid tumour progression caused by HIV-induced immunodeficiency. (Myers and Ahmed, 2019) This hypothesis is consistent with our finding that HIV-positive patients were less likely to receive surgery and more likely to receive radiotherapy or chemotherapy than HIV-negative patients. Surgery is indicated for early-stage cervical cancer, whereas chemotherapy is mainly used for advanced disease. Interestingly, the percentage of women with metastases at diagnosis did not differ by HIV status in our analysis. Another explanation for differences in survival by HIV status could be the detrimental effect of HIV on overall health, resulting in higher rates of non-cancer mortality in HIV-positive patients. ART has greatly improved life expectancy among WLWH, (Trickey et al., 2017) but only 67 % of our HIV-positive cervical cancer patients were on ART at cancer diagnosis. Some studies have suggested that immunodeficiency might interfere with the ability to tolerate chemotherapy and complete adequate cancer treatment, while others found no differences. (Shah et al., 2021, Einstein et al., 2019).

In conclusion, we found that the risk of death was higher in HIV-positive than HIV-negative cervical cancer patients who received cancer treatment in South Africa. A better understanding of differences in tumour progression, clinical care, and HIV-specific mortality that may explain this finding is needed. However, the poor survival in our study also highlights the importance of detecting and treating cervical lesions before cervical cancer can develop, particularly among WLWH.

## Author contributions

YQT, YR, and ER conceptualized the study; ADH, YQT, and YR were involved in the data management; YR performed the data analysis; YQT and ER wrote the first draft of the manuscript; all authors contributed to the interpretation of the results, reviewed the manuscript, and agreed with the final version.

## Declaration of Competing Interest

TMGB is on the advisory boards of Discovery Health Medical Scheme and serves as a senior medical advisor on HIV within Aid for AIDS/Medscheme. The other authors declare that they have no known competing financial interests or personal relationships that could have appeared to influence the work reported in this paper.
