# Supplementary material for: Cancer treatment and survival among cervical cancer patients living with or without HIV in South Africa
Source: Gynecol Oncol Rep. 2022 Sep 21;43:101069. doi: 10.1016/j.gore.2022.101069 (PMC9516451; doi:10.1016/j.gore.2022.101069)

**Supplementary Table S1: Reimbursement claims codes used for definition of variables and patient selection.**

| Definition | Code Type | Codes |
|---|---|---|
| Cervical Cancer | ICD-10 | C53 - C53.9 |
| HIV | ICD-10 | B20-B24 |
| ART | ATC | J05AE, J05AF, J05AG, J05AJ, J05AR |
| Cancer stage | ICD-10 | C77-C79.9 |
| | ICD-O-3 | M8000/6, M8010/6, M8070/6, M8140/6, M8230/6 |
| Squamous histology | ICD-O-3 | M8051/3, M8052/3, M8070/3, M8071/3, M8072/3, M8076/3, M8082/3, M8083/3, M8120/3 |
| Glandular histology | ICD-O-3 | M8140/3, M8144/3, M8262/3, M8310/3, M8380/3, M8441/3, M8480/3, M8482/3, M8490/3, M9110/3 |
| Other histology | ICD-O-3 | M8013/3, M8015/3, M8020/3, M8041/3, M8098/3, M8200/3, M8240/3, M8249/3, M8560/3, M8570/3, M8720/3, M8805/3, M8890/3, M8910/3, M8931/3, M8980/3, M8933/3, M8960/3, M9071/3, M9120/3, M9540/3, M9581/3 |
| Radiotherapy | ICD-10 | Z51.0 |
| | NRPL | 5635-5861, 5882-5893, 88801-88819 |
| | CPT | 57155, 57156, 58346, 77014, 77295-77620, 77750-77799, 79005, 79101, 79403 |
| Chemotherapy | ICD-10 | Z51.1, Z51.2 |
| | CPT | 96413-96416 |
| | NRPL | 5790-5795, 88851, 88853-88855, 99951, 99953-99955, 99959 |
| | ATC | L01XA01, L01XA02, L01CD01, L01CE01, L01CD02, L01AA06, L01BC02, L01CE02, L01BC05, L01DC03, L01XC07, L01XC18, L01BA04, L01CA04 |
| Surgery | CPT | 57530-57556, 58150, 58152, 58200-58294, 58548-58554, 58570-58575 |
| | NRPL | 2343, 2345, 2349, 2357, 2407, 2471, 2473, 2475, 2532 |

**Supplementary Table S2: Unadjusted odds ratios for receiving cancer treatment within 6 months after cervical cancer diagnosis.**

| Characteristic | Patients treated (N) | OR for radiotherapy (95% CI) | Patients treated (N) | OR for chemotherapy (95% CI) | Patients treated (N) | OR for surgery (95% CI) |
|---|---|---|---|---|---|---|
| **HIV status** | | | | | | |
| Negative | 236 | 1 | 284 | 1 | 121 | 1 |
| Positive | 113 | 2.31 (1.42-3.89) | 125 | 2.52 (1.33-5.2) | 29 | 0.51 (0.31-0.8) |
| **Age category** | | | | | | |
| <40 years | 63 | 1 | 73 | 1 | 38 | 1 |
| 40-59 years | 203 | 1.27 (0.75-2.13) | 238 | 1.63 (0.86-3.01) | 81 | 0.59 (0.36-0.96) |
| ≥60 years | 83 | 1.05 (0.58-1.91) | 98 | 1.21 (0.59-2.45) | 31 | 0.50 (0.28-0.89) |
| **Ethnicity*** | | | | | | |
| Black | - | 1 | - | 1 | - | 1 |
| Other** | - | 0.80 (0.51-1.28) | - | 0.61 (0.36-1.05) | - | 1.80 (1.16-2.77) |
| **Histological tumour type*** | | | | | | |
| Squamous cell carcinoma | - | 1 | - | 1 | - | 1 |
| Adenocarcinoma | - | 0.24 (0.14-0.42) | - | 0.42 (0.21-0.84) | - | 1.58 (0.91-2.73) |
| Other | - | 0.18 (0.07-0.45) | - | 0.16 (0.06-0.44) | - | 2.57 (1.04-6.35) |
| **Cancer stage** | | | | | | |
| Localised | 318 | 1 | 361 | 1 | 141 | 1 |
| Metastasised | 31 | 0.52 (0.29-0.96) | 48 | 2.33 (0.91-7.89) | 9 | 0.43 (0.19-0.87) |

* Missing values for ethnicity and histological tumour type were imputed using multiple imputation.

** Other include White, Indian/Asian, and Mixed ancestry ethnicities.

**Supplementary Table S3: Odds ratios for receiving cancer treatment within 6 months after cervical cancer diagnosis, including separate categories for patients with missing values on ethnicity and histological tumour type (sensitivity analysis).**

| Characteristic | Patients treated (N) | Univariable | Multivariable | Patients treated (N) | Univariable | Multivariable | Patients treated (N) | Univariable | Multivariable |
|---|---|---|---|---|---|---|---|---|---|
| | | OR for radiotherapy (95% CI) | | | OR for chemotherapy (95% CI) | | | OR for surgery (95% CI) | |
| **HIV status** | | | | | | | | | |
| Negative | 236 | 1 | | 284 | 1 | 1 | 121 | 1 | 1 |
| Positive | 113 | 2.31 (1.42-3.89) | 1.91 (1.08-3.47) | 125 | 2.52 (1.33-5.2) | 1.96 (0.93-4.43) | 29 | 0.51 (0.31-0.8) | 0.5 (0.29-0.84) |
| **Age category** | | | | | | | | | |
| <40 years | 63 | 1 | 1 | 73 | 1 | 1 | 38 | 1 | 1 |
| 40-59 years | 203 | 1.27 (0.75-2.13) | 1.44 (0.81-2.54) | 238 | 1.63 (0.86-3.01) | 1.86 (0.93-3.68) | 81 | 0.59 (0.36-0.96) | 0.57 (0.34-0.96) |
| ≥60 years | 83 | 1.05 (0.58-1.91) | 1.74 (0.88-3.46) | 98 | 1.21 (0.59-2.45) | 1.59 (0.71-3.55) | 31 | 0.5 (0.28-0.89) | 0.37 (0.19-0.7) |
| **Ethnicity** | | | | | | | | | |
| Black | 209 | 1 | 1 | 243 | 1 | 1 | 82 | 1 | 1 |
| Other* | 81 | 0.78 (0.49-1.24) | 1.04 (0.62-1.75) | 92 | 0.59 (0.34-1.02) | 0.8 (0.44-1.46) | 51 | 1.86 (1.19-2.9) | 1.49 (0.92-2.43) |
| Unknown | 59 | 1.07 (0.61-1.93) | 0.97 (0.53-1.84) | 74 | 2.56 (1.06-7.61) | 2.26 (0.9-6.93) | 17 | 0.68 (0.37-1.21) | 0.77 (0.41-1.41) |
| **Type of tumour** | | | | | | | | | |
| Squamous cell carcinoma | 190 | 1 | 1 | 206 | 1 | 1 | 59 | 1 | 1 |
| Adenocarcinoma | 34 | 0.24 (0.12-0.48) | 0.27 (0.13-0.56) | 45 | 0.27 (0.1-0.7) | 0.31 (0.12-0.84) | 21 | 1.7 (0.9-3.16) | 1.69 (0.85-3.32) |
| Other | 8 | 0.19 (0.06-0.61) | 0.2 (0.06-0.67) | 10 | 0.13 (0.04-0.55) | 0.14 (0.04-0.57) | 6 | 2.01 (0.64-6.02) | 2.07 (0.63-6.54) |
| Unknown | 117 | 0.21 (0.12-0.33) | 0.22 (0.13-0.37) | 148 | 0.16 (0.08-0.3) | 0.17 (0.08-0.33) | 64 | 1.28 (0.84-1.95) | 1.16 (0.75-1.82) |
| **Cancer stage** | | | | | | | | | |
| Localised | 318 | 1 | 1 | 361 | 1 | 1 | 141 | 1 | 1 |
| Metastasised | 31 | 0.52 (0.29-0.96) | 0.59 (0.31-1.12) | 48 | 2.33 (0.91-7.89) | 2.77 (1.05-9.6) | 9 | 0.43 (0.19-0.87) | 0.42 (0.18-0.87) |

* Other include: (radiotherapy) 52 White, 14 Indian/Asian, 15 Mixed ancestry; (chemotherapy) 60 White, 15 Indian/Asian, 17 Mixed ancestry; (surgery) 35 White, 7 Indian/Asian, 9 Mixed ancestry

**Supplementary Table S4: Unadjusted and adjusted hazard ratios for all-cause mortality after cervical cancer diagnosis, including separate categories for patients with missing values on ethnicity and histological tumour type (sensitivity analysis).**

| Characteristic | Deaths (N) | Univariable Hazard Ratio (95% CI) | Multivariable Hazard Ratio (95% CI) |
|---|---|---|---|
| **HIV status** | | | |
| Negative | 149 | 1 | 1 |
| Positive | 63 | 1.18 (0.88-1.58) | 1.57 (1.1-2.24) |
| **Age category** | | | |
| <40 years | 36 | 1 | 1 |
| 40-59 years | 105 | 1.07 (0.73-1.56) | 1.13 (0.77-1.66) |
| ≥60 years | 71 | 2.01 (1.35-3.02) | 2.34 (1.49-3.66) |
| **Ethnicity** | | | |
| Black | 132 | 1 | 1 |
| Other* | 43 | 0.72 (0.51-1.01) | 0.73 (0.51-1.06) |
| Unknown | 37 | 1.03 (0.71-1.48) | 0.97 (0.66-1.4) |
| **Type of tumour** | | | |
| Squamous cell carcinoma | 78 | 1 | 1 |
| Adenocarcinoma | 30 | 1.52 (1-2.32) | 1.27 (0.81-1.99) |
| Other | 8 | 2.03 (0.98-4.21) | 2.05 (0.98-4.3) |
| Unknown | 96 | 1.29 (0.96-1.74) | 1.35 (0.99-1.84) |
| **Cancer stage (mortality up to 9 months)** | | | |
| Localised | 67 | 1 | 1 |
| Metastasised | 27 | 4.37 (2.79-6.84) | 3.78 (2.38-6.00) |
| **Cancer stage (mortality after 9 months)** | | | |
| Localised | 107 | 1 | 1 |
| Metastasised | 11 | 1.54 (0.83-2.87) | 1.37 (0.72-2.57) |

* Other include 80 White, 18 Indian/Asian, 21 Mixed ancestry

The adjusted hazard ratios for HIV status, age, and ethnicity, are obtained from a single model stratified with respect to the cancer stage variable. The hazard ratios (both adjusted and unadjusted) for cancer stage are obtained from separate models, one censoring time-at-risk at 9 months, and another starting time-at-risk at 9 months.

**Supplementary Figure S1: Kaplan-Meier (Panel A) and age-standardized overall survival curves (Panel B), stratified by cancer stage.**

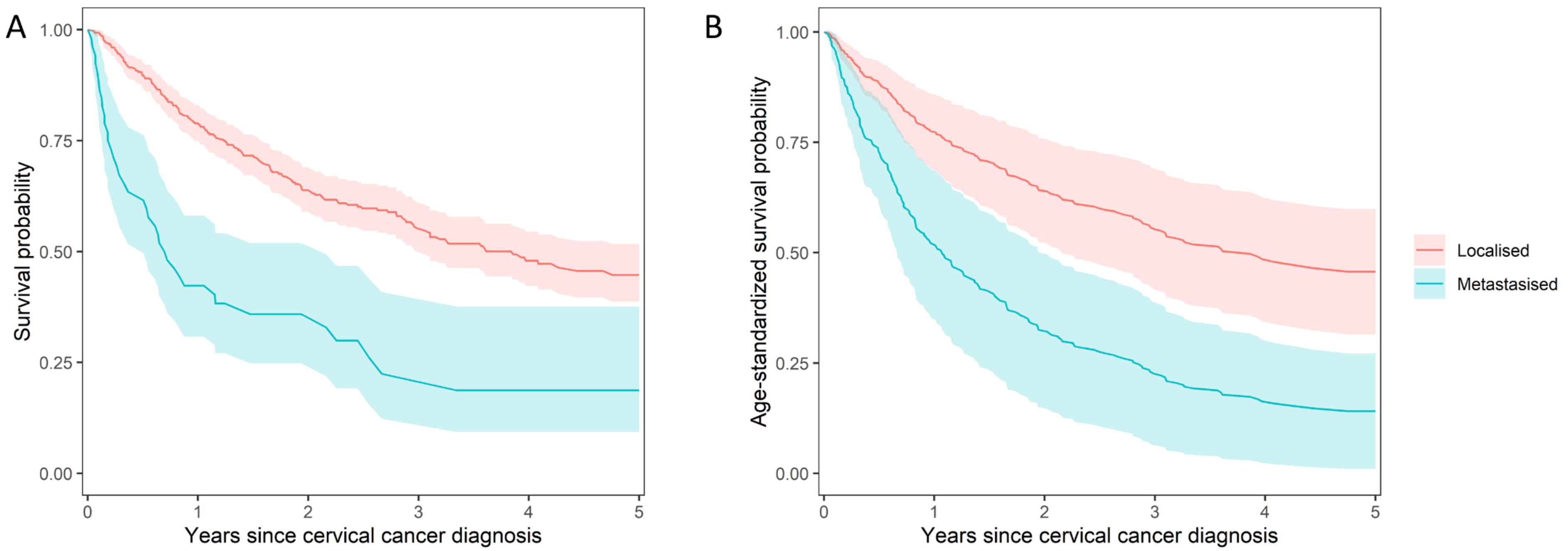

**Supplementary Figure S2: Kaplan-Meier (Panel A) and age-standardized overall survival curves (Panel B), stratified by HIV and ART status.**

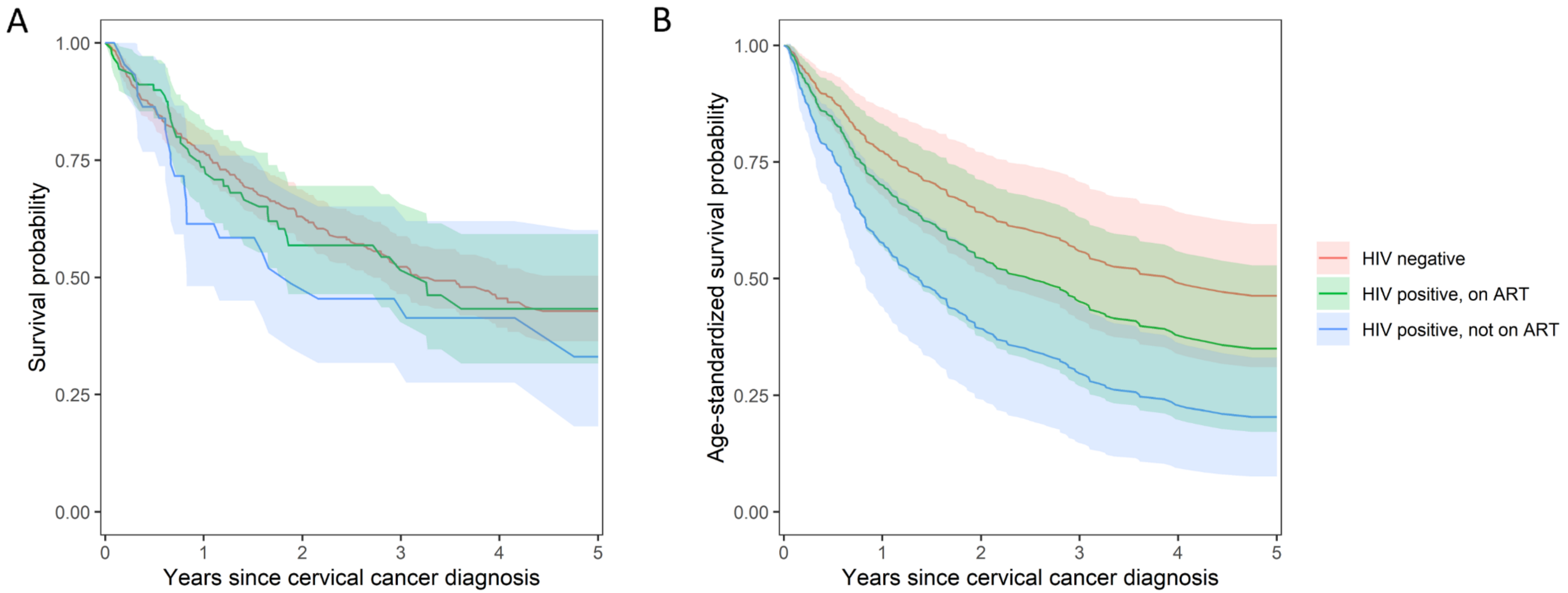

Supplement: Supplementary data 1 [file mmc1.pdf]
